# Short Tandem Repeat (STR) DNA Analysis for Using Coffee Cups As Forensic Medicine Evidence

**DOI:** 10.7759/cureus.47592

**Published:** 2023-10-24

**Authors:** Abdullah Saeed, Abdullah AlShafea, Faisal A AlFaya, Mohammed Y Asiri, Abdulrahman Bin Saeed, Ali Alnasser

**Affiliations:** 1 Action Research, Ministry of Health, Abha, SAU; 2 Research Unit, Ministry of Health, Abha, SAU; 3 Primary Healthcare, Ministry of Health, Abha, SAU; 4 Research and Studies, Health Affairs Aseer Region, Abha, SAU; 5 Public Health, King Abdulaziz University, Abha, SAU

**Keywords:** genetic screening, dna-seq, forensic medicine, clinical forensic medicine, short tandem repeat

## Abstract

Introduction

Forensic science has continually evolved, with innovations in DNA analysis techniques playing a pivotal role in improving the accuracy and reliability of criminal investigations. Short tandem repeats (STRs) have been a game-changer in forensic science, offering unique genetic markers to pinpoint individual identities. This study explores the application of STR DNA analysis to an unconventional source: coffee cups.

Materials and methods

In a study involving 16 unrelated, healthy individuals aged 26 to 32, DNA was investigated from coffee cups and mouth cavities, comparing the results to a previous coffee cup DNA study. Buccal swabs provided reference samples, air-dried for 10 minutes, and stored at 20°C. DNA quantification revealed a significant decrease in coffee cup samples (0.015 to 0.049 ng/μL) compared to the reference, with no DNA in negative controls. Some STR loci displayed inhibition and lower heterozygosity in the research samples. Ethical approval (REC 53-592) and adherence to the Declaration of Helsinki ensured ethical standards. This research highlights potential challenges in obtaining efficient DNA profiles from various sources.

Results

There was a significant variation in DNA concentrations among the different cup types, with ceramic cups yielding the highest concentrations. Moreover, the DNA profiling outcomes ranged from full profiles, which allow for precise individual identification, to partial profiles, which pose challenges for conclusive matches. These findings have profound implications for forensic science. The study demonstrates the potential of coffee cups as sources of DNA evidence in criminal investigations, even if partial profiles are obtained. Such evidence can assist in suspect identification, victim profiling, and corroborating witness statements.

Conclusion

This study highlights the application of STR DNA analysis for coffee cups, opening new avenues for forensic investigations and highlighting the need for continued research and development in this exciting field. This observation aligns with existing research on DNA recovery from various surfaces, and further research is warranted to refine the methodology, consider usage patterns, and address ethical and legal concerns regarding the collection and use of DNA evidence from common objects.

## Introduction

The identification of DNA STR profiles has revolutionized forensic science, enabling the accurate and reliable determination of an individual's genetic information [1]. Short tandem repeats (STRs) are specific regions of DNA that contain repeating sequences of nucleotides. These regions are highly variable among individuals, making them ideal for forensic analysis [2]. Using STR profiling in criminal investigations has significantly improved the accuracy of identifying suspects and exonerating innocent individuals. Furthermore, STR profiling has also played a crucial role in resolving cold cases that have remained unsolved for years [3]. By comparing the STR profiles of crime scene evidence with DNA samples from potential suspects, investigators can establish links between individuals and crime scenes with remarkable certainty [4]. This has not only led to the successful prosecution of criminals but also brought closure to the families of victims who have long awaited justice. In addition, STR profiling has helped identify wrongful convictions, allowing innocent individuals to be freed from prison and their names to be cleared [5]. The power of this technique lies in its ability to accurately match DNA samples to individuals, providing solid evidence that can withstand scrutiny in a court of law. This has revolutionized the field of forensic science and has become an invaluable tool for law enforcement agencies worldwide [6]. Furthermore, STR profiling has significantly reduced the chances of mistaken identity, ensuring that the right person is held accountable for their actions [7-8]. The impact of STR profiling on criminal investigations cannot be overstated, as it has transformed how cases are solved and how justice is served. For example, STR profiling can analyze DNA samples found at the crime scene and compare them to those of potential suspects [9]. By matching specific genetic markers, forensic scientists can provide strong evidence linking an individual to the crime. This scientific method has helped convict criminals who would have otherwise gone unpunished and exonerate innocent individuals who were wrongly accused. In one notable case, STR profiling was crucial in identifying and convicting a serial killer who had eluded law enforcement for years [10]. The DNA evidence collected from various crime scenes was compared to a database of known offenders, and a match was found [11]. The power of DNA analysis in criminal investigations cannot be understated as it continues to revolutionize the field of forensic science and contributes to the pursuit of truth and justice. In addition to identifying perpetrators, DNA analysis has also played a crucial role in exonerating wrongfully convicted individuals. Countless innocent lives have been saved from wrongful imprisonment because of advancements in this technology. Furthermore, DNA evidence has helped solve cold cases that had stumped investigators for years, offering solace to families who had long given up hope of finding answers [12].

The ability to extract valuable information from the slightest DNA traces has transformed how crimes are investigated and prosecuted. In addition to its role in solving crimes, DNA analysis has also found a valuable application in tracing the origins of biological evidence found in cups. It can be extended to other similar surfaces. By carefully examining the DNA present on the surface of a cup, forensic scientists can gather crucial information about the person who may have used it. This includes their gender, ethnicity, and even potential familial relationships. Such insights can be instrumental in narrowing down a list of suspects or identifying a potential perpetrator. The ability to analyze trace amounts of DNA on cups has opened up new avenues for forensic investigations, allowing law enforcement agencies to gather vital clues and build stronger cases. By studying the DNA, scientists can determine a person's genetic markers, providing information about their physical traits and ancestry [13]. This can help investigators create a detailed suspect profile, aiding in the identification process. Overall, DNA analysis on cups has revolutionized forensic investigations, providing valuable evidence that can lead to justice being served [14].

## Materials and methods

The study included a group of 16 men and women who were all in good health and were not related to one another in any manner. The participants holding the coffee and drinking it without being blinded ranged in age from 26 to 32 years, with an average age of 28. The study team took an oral cavity DNA sample after obtaining informed consent. This study aimed to investigate the presence of DNA on different types of coffee cups, which are often used in a wide variety of coffee shops. DNA within the mouth cavities of the same persons who had contributed was used as reference samples. This study aimed to understand better the possibility of cross-contamination across different kinds of coffee cups.

The most popular cups were identified and gathered for the research after using a survey distributed randomly at coffee shops to collect information on regularly used coffee cups. This was done so that we could put this knowledge to use at some point in the selection of cups. In addition, the DNA trace in the most well-known types of coffee cups was examined as part of the research project so that the researchers could investigate any potential sources of contamination.

To establish reference samples that could be compared with the DNA extracted from coffee cups and other objects connected to coffee cups, buccal swabs were used to swab the mouth cavities of each of the 16 donors. This allowed for comparisons to be made between the two sets of DNA. Following that, the DNA that had been isolated from coffee cups was compared to these samples. These buccal swabs were given 10 minutes to air-dry at room temperature before extracting their DNA. This step occurred before the extraction process. Following that, DNA from both cups and swabs was stored in a facility where the temperature was rigorously controlled and maintained at 20 degrees Celsius.

A method referred to as 7500 real-time PCR (qRT-PCR), which stands for quantitative real-time PCR, was utilized to quantify the amount of DNA present. A set of 16 STR loci was targeted to amplify the collected DNA. This was accomplished with the assistance of the AmpFlSTR® Identifiler® PCR amplification kit (Applied Biosystems, Massachusetts, USA) and the ABI Geneamp 9700 PCR thermocycler (Applied Biosystems, Massachusetts, USA). The purpose of this was to attain the necessary amount of amplification. This process increased the total amount of DNA in the sample. After the PCR products were created, they were analyzed with a 3130 genetic analyzer (Applied Biosystems, Massachusetts, USA) and the Gene Mapper® ID-X software version 3.5 (Applied Biosystems, Massachusetts, USA).

The results of the DNA quantification showed a significant decrease in the quantity of DNA present in the samples obtained from coffee cups compared to the reference samples. This was demonstrated by the fact that there was a significant difference between the two sets of samples in the amount of DNA. The amounts of DNA found in the samples taken from coffee cups ranged from 0.015 to 0.049 nanograms per microliter (ng/L). These findings were expressed as numerical values. When compared to both the reference and the negative control samples, particular STR loci exhibited significant inhibition and reduced the amount of heterozygosity seen in the study samples. In addition, the heterozygosity of the samples included in the study was lower. This provides evidence that there may be difficulties in getting DNA profiles from various sources that may be utilized efficiently. The results of this investigation were compared to those of previous studies that investigated the presence of DNA on coffee cups.

This study was carried out with the utmost regard for ethical standards that were specified in the Declaration of Helsinki in 1964, as well as any future revisions to those guidelines that may have been made, as shown by the fact that it was approved by the National Research Ethics Committee and assigned the registration number REC 53-592. This paper provides an in-depth explanation of the ethical goals and methods of the research project, ensuring that the individuals who participated in the study will be treated with kindness and their legal entitlements will be respected.

## Results

**Figure 1:**
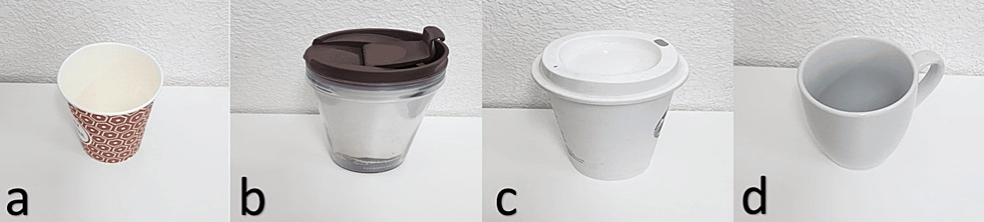
Different cups used in the study (a) Carton cup, (b) travel mug, (c) plastic cup, and (d) ceramic cup

The extracted DNA concentrations ranged from as low as 0.0166 ng/L to as high as 0.0485 ng/L in these cups. In addition, the DNA profiling findings were inconsistent, with some cups producing full profiles, indicating a complete genetic profile, and others producing partial profiles, indicating an incomplete genetic profile. This study demonstrates the possibility of retrieving DNA evidence from several types of cups (Figure 1), which can be useful for forensic investigations. The effectiveness of getting a complete or partial DNA profile appears to be affected by both the kind of cup and the amount of DNA on the cup's surface. This study provides new opportunities for forensic science and criminal investigations by demonstrating the viability of employing cups as a possible source of DNA evidence (Table 1).

**Table 1:** DNA concentration in different cups

Serial no	Cups type	DNA concentration (ng/μL)	DNA profiling (no. of STR loci obtained)
1	Carton cup	0.0166	Partial profile
2	Plastic cup	0.0341	Full profile
3	Travel mug	0.0221	Partial profile
4	Ceramic cup	0.0485	Full profile
5	Carton cup	0.0166	Partial profile
6	Plastic cup	0.0230	Partial profile
7	Travel mug	0.0299	Partial profile
8	Ceramic cup	0.0336	Full profile
9	Carton cup	0.0329	Full profile
10	Plastic cup	0.0305	Full profile
11	Travel mug	0.0403	Full profile
12	Ceramic cup	0.0395	Full profile
13	Carton cup	0.0392	Full profile
14	Plastic cup	0.0191	Partial profile
15	Travel mug	0.0235	Partial profile
16	Ceramic cup	0.0322	Full profile

**Figure 2:**
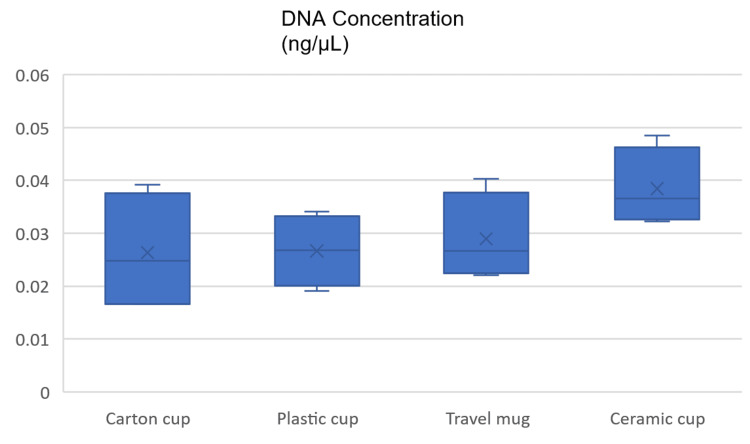
Box plot showing DNA concentration (ng/μL) in different cups

The table summarizes DNA analysis results for different cup types. Ceramic cups yielded the highest average DNA concentration (0.0384 ng/μL) and produced the most full profiles (4). Travel mugs had slightly lower DNA concentration (0.0289 ng/μL) but exhibited the least variability. Carton and plastic cups had similar DNA concentrations (around 0.0263-0.0266 ng/μL) and generated two full profiles each. Standard deviations ranged from 0.003 to 0.006 ng/μL. These findings represent that ceramic cups are most appropriate for DNA analysis due to their higher concentration and reliability, while travel mugs also perform well with lower variability (Table 2, Figure 2).

**Table 2:** Mean and SD of DNA concentration in different cups

Cups type	DNA concentration (ng/μL)	Standard deviation (ng/μL)	Full profile count
Carton cup	0.0263	0.005	2
Plastic cup	0.0266	0.004	2
Travel mug	0.0289	0.003	2
Ceramic cup	0.0384	0.006	4

## Discussion

The results of this study reveal valuable insights into the feasibility of obtaining DNA from various types of cups, shedding light on the potential applications and limitations of this innovative forensic approach. These findings have significant implications in the field of forensic science and criminal investigations.

DNA concentration variation

The observed variations in DNA concentrations among different types of cups align with previous research on DNA recovery from metal surfaces [15]. Ceramic cups, with their porous surfaces, seem to be more conducive to retaining DNA material in higher concentrations. In contrast, carton and plastic cups may not retain DNA as effectively due to their non-porous surfaces. These results are consistent with studies investigating DNA recovery from different materials and surfaces [16].

DNA profiling outcomes

The categorization of DNA profiles into full and partial profiles underscores the importance of DNA quantity and quality. Complete profiles obtained from cups provide a valuable source of genetic information that can be used to identify individuals with a high degree of certainty. However, partial profiles raise concerns about the reliability of matching DNA evidence to specific individuals. Partial profiles could result from factors such as low DNA quantity or degradation, as observed in previous studies [17].

Forensic implications

The ability to recover DNA evidence from cups, even in the form of partial profiles, has substantial forensic implications. This study highlights the potential for cups to serve as valuable sources of DNA evidence in criminal investigations, aiding in suspect identification, victim profiling, and corroborating witness statements. Similar findings have been reported in studies involving the recovery of DNA from common objects like drinking glasses [18].

Future research and considerations

While this study provides a foundation for using cups as potential sources of DNA evidence, further research is essential to refine the methodology. Future investigations should consider factors such as cup usage patterns, environmental conditions, and the impact of time on DNA recovery. Additionally, ethical and legal considerations surrounding collecting and using DNA evidence from common objects like cups must be addressed, as discussed in previous studies [19].

Expanding on the discussion of the results, it's important to consider the implications of the findings beyond just the type of cup and DNA concentration. The variability in DNA recovery across different cup types underscores the need for a nuanced approach in forensic investigations. Here are some additional points to consider:

Environmental Factors

Environmental conditions, such as humidity, temperature, and contaminant exposure, may also influence the study results. These factors could affect the stability of DNA on cup surfaces and its subsequent recovery.

Time Sensitivity

DNA degradation over time is a critical factor. The study did not assess the duration between when the cups were used and when samples were collected. This is important because the longer a cup has been used, the more likely DNA might degrade or be washed away.

Handling and Storage

The handling and storage of cups, as well as the collection process, can introduce potential sources of contamination. Ensuring proper protocols are followed during sample collection and preservation is essential for maintaining the integrity of the evidence.

Forensic Significance

While the study demonstrates the presence of DNA on cups, evaluating the forensic significance of partial profiles is crucial. Partial profiles, as seen in carton and plastic cups, can still provide valuable information but might not be as conclusive as complete profiles. The extent to which partial profiles can be used in casework should be explored further.

Population Variability

The study involved a small sample of men and women from the same geographical region. The effectiveness of DNA retrieval from cups may vary across different populations and geographical regions due to genetic diversity and dietary habits. A broader study with a more diverse sample could provide a more comprehensive understanding.

Trace Evidence

In forensic cases, trace evidence can be pivotal. Even low DNA concentrations, as seen in some cup types, may be sufficient for a match or investigative lead. The study could be expanded to determine the minimum detectable DNA concentration for forensic utility.

Legal and Ethical Considerations

Using cups as a source of DNA evidence raises legal and ethical questions. Privacy concerns, the chain of custody, and the admissibility of such evidence in court should be addressed.

Forensic Best Practices

Law enforcement and forensic experts must establish best practices for collecting, preserving, and analyzing DNA from cups and similar items. This includes standardized protocols, quality control measures, and validation studies to ensure the reliability of results.

This study provides a foundation for understanding the potential for retrieving DNA evidence from cups, which can be valuable in forensic investigations. However, it also highlights the need for further research, encompassing a broader range of cup types and environmental conditions, and the development of standardized procedures for DNA recovery and analysis. The collaboration between forensic scientists, geneticists, and legal experts is crucial in harnessing the full potential of this emerging avenue of forensic evidence.

The study has several limitations. Firstly, the sample size is relatively small, comprising 16 participants from a specific age group and geographic region, which may not represent the broader population. Additionally, the study does 100% control for various environmental factors that could affect DNA recovery, such as humidity and exposure to contaminants. The lack of information on the duration between cup use and sample collection introduces uncertainty about DNA degradation for future studies.

## Conclusions

This work contributes to the developing body of knowledge in the burgeoning field of research that focuses on collecting DNA evidence from cups. The topic of this research is currently receiving a lot of attention. Recently, a lot of emphasis has been focused on this topic. The findings suggest that cups can potentially be valuable sources of genetic information in forensic investigations, which could ultimately lead to breakthroughs in the field of criminal justice. Cups can potentially be useful sources of genetic information in forensic investigations. However, greater research and validation are necessary to develop best practices and overcome any limitations associated with this one-of-a-kind approach to forensic science.
